# Observations in European Surgery

**Published:** 1896-05

**Authors:** 


### OBSERVATIONS IN EUROPEAN SURGERY.

Professor Keen, of Jefferson Medical College, spent a considerable part of last year visiting the various European hospitals, and on his return gave some observations on surgical work abroad. His lecture is interesting and instructive. It is especially valuable, too, because Professor Keen is a most painstaking, able surgeon, and is, therefore, a proper person to point out differences which are genuinely distinctive.

He was pleased to notice the great change which had come over the minds of foreign surgeons as regards the estimate in which American medicine is held. Formerly the work of American medical men was regarded lightly. The opinion prevailed that all the essentials to correct observation was lacking in the American surgeon. Dr. Keen was pleased to see that this view was now radically changed, and that American medical magazines, and American works on the various departments of medical inquiry were in all libraries, and on the tables of physicians everywhere.

Professor Keen is convinced that European surgeons are very much in advance of us, and that they are more original. He attributes this to the longer course of study required by the medical student, and to the government support of pathological and bacteriological institutions, and to certainty to which well-qualified men can look for honorable and lucrative preferment.

Professor Keen, however, declares that American surgeons are not eclipsed anywhere in the quality of practicability. He regards the European as far more careful as in matters of antiseptia. A feature which struck Professor Keen was that the European surgeon washed his hands *longer* than is commonly done in our country. Professor Keen resolved that in the future he would consume all the time that he could before an operation in washing his hands! In America it is the custom of only going through the motion of washing one's hands! The editor has no desire to underrate the cleanliness of American surgery, which is excellent, but it is true a great deal of the washing of hands could be done on the European principle with improvement!

Another feature which struck Professor Keen as most distasteful and outrageous was the needless exposure of person to which patients were subjected to in the hospital wards. Men and women were brought out before classes almost entirely nude, when it was altogether unnecessary. This is one of the shameful practices of European hospitals which has been noticed by other American travelers in Europe.

The existing practice of European surgeons in giving anæsthetics is almost reckless. The chloroform is poured in a napkin or towel and pressed close to the patient's face, so as to allow almost no air. In six weeks Dr. Keen saw no less than six patients brought almost to death by this manner of the administration of chloroform. With this mode of the administration of anæsthetics Dr. Keen clearly sees why so many deaths are reported from Europe from anæsthetics.

Dr. Keen was invited to attend the examination of the students of The Royal College of Surgeons. They are described as thorough and much superior to those in America.—*Medical Progress.*

